# Structural and functional diversity among Type III restriction-modification systems that confer host DNA protection via methylation of the N4 atom of cytosine

**DOI:** 10.1371/journal.pone.0253267

**Published:** 2021-07-06

**Authors:** Iain A. Murray, Yvette A. Luyten, Alexey Fomenkov, Nan Dai, Ivan R. Corrêa, William G. Farmerie, Tyson A. Clark, Jonas Korlach, Richard D. Morgan, Richard J. Roberts

**Affiliations:** 1 New England Biolabs, Ipswich, Massachusetts, United States of America; 2 Interdisciplinary Center for Biotechnology Research, University of Florida, Gainesville, Florida, United States of America; 3 Pacific Biosciences Inc., Menlo Park, California, United States of America; Universität Stuttgart, GERMANY

## Abstract

We report a new subgroup of Type III Restriction-Modification systems that use m4C methylation for host protection. Recognition specificities for six such systems, each recognizing a novel motif, have been determined using single molecule real-time DNA sequencing. In contrast to all previously characterized Type III systems which modify adenine to m6A, protective methylation of the host genome in these new systems is achieved by the N4-methylation of a cytosine base in one strand of an asymmetric 4 to 6 base pair recognition motif. Type III systems are heterotrimeric enzyme complexes containing a single copy of an ATP-dependent restriction endonuclease-helicase (Res) and a dimeric DNA methyltransferase (Mod). The Type III Mods are beta-class amino-methyltransferases, examples of which form either N6-methyl adenine or N4-methyl cytosine in Type II RM systems. The Type III m4C Mod and Res proteins are diverged, suggesting ancient origin or that m4C modification has arisen from m6A MTases multiple times in diverged lineages. Two of the systems, from thermophilic organisms, required expression of both Mod and Res to efficiently methylate an *E*. *coli* host, unlike previous findings that Mod alone is proficient at modification, suggesting that the division of labor between protective methylation and restriction activities is atypical in these systems. Two of the characterized systems, and many homologous putative systems, appear to include a third protein; a conserved putative helicase/ATPase subunit of unknown function and located 5’ of the *mod* gene. The function of this additional ATPase is not yet known, but close homologs co-localize with the typical Mod and Res genes in hundreds of putative Type III systems. Our findings demonstrate a rich diversity within Type III RM systems.

## Introduction

The Type III restriction enzymes EcoPI and EcoP15I—originally isolated from coliphage P1 and *E*.*coli* plasmid P15, respectively—are among the earliest examples of RM systems to be identified and characterized [1, 2]. Type III systems are typically composed of two functionally distinct proteins, Mod and Res. The Mod subunit is responsible for specific DNA sequence recognition and host-protective DNA methylation. The Res subunit contains the endonuclease domain for DNA cleavage and an ATP hydrolysis domain related to SF2 helicases. Restriction requires both mod and res subunits, whereas methylation generally requires only the mod enzyme. In all previously known systems, the mod subunit confers host DNA protection via N6-methylation of a deoxyadenosine base located within just one strand of a non-palindromic target DNA sequence. The protein sequences of the EcoPI and EcoP15I Res subunits are almost identical and the corresponding Mod subunits are also closely related. However, the amino acid sequences encoding the specificity-defining target recognition domains (TRD) within each of the Mod subunits are divergent. M.EcoPI methylates the adenine base at the third position of 5’-AGACC-3’ sequences and M.EcoP15I methylates the adenine base at the fifth position of 5’-CAGCAG-3’ sequences [3, 4]. In the absence of cognate methylation, substrate DNAs are restricted by a Mod-Res complex in a reaction that requires ATP hydrolysis and the presence of two or more recognition sites arranged in an inverse orientation—either head-to-head or tail-to-tail [5–8]. Initial models of Type III enzymes—invoking a heterotetrameric structure comprised of two Mod and two Res subunits per active complex [9]—were subsequently revised in favor of a heterotrimeric Mod-2/Res-1 architecture [10, 11]. Structural analyses of EcoP15I—using small-angle X-ray scattering [12] and X-ray crystallography [13]—are also consistent with a single Res subunit associated with a Mod homodimer. In the crystal structure one subunit of the Mod dimer appears to be primarily responsible for DNA binding while the second catalyzes methylation of the adenine at the 5th position in the EcoP15I target sequence. Res-catalyzed phosphodiester bond hydrolysis of unmethylated substrates typically yield products with two base-pair (bp) 5’-extensions at a position 25 and 27 base-pairs 3’ of the Mod motif [3, 4]. Several additional Type III RM systems have been characterized, including HinfIII which methylates the second A in the sequence 5’-CGAAT-3’, PstII which methylates the A at the fourth position in the sequence 5’-CTGATG-3’, and BceSI that methylates the A at the fourth position of the sequence 5’-CGAAG-3 [14–16]. However, in contrast to the Type II RM systems that provided the critical tools for the development of recombinant DNA technologies, relatively few Type III RM systems have been characterized in detail. This reflects the difficulty of characterizing cleavage sites by the Res component, which often gives incomplete cleavage. In addition, the analyses typically required to define the sequence motifs recognized by Mod subunits—and the identity of the specific A that was methylated—were technically challenging. However, with the introduction of Single Molecule Real Time (SMRT) sequencing technology it is now possible to identify N6-methyldeoxyadenosine (m6A) and N4-methyldeoxycytosine (m4C) modifications on a genome-wide scale thereby providing a direct readout of Mod specificities [17–19].

Based on SMRT sequencing observations of asymmetric, single strand m4C modification motifs in organisms that harbor putative Type III RM systems, we characterized the recognition specificity for six Type III systems that use m4C methylation for host protection and confirmed endonuclease cleavage typical of Type III enzymes for one of these systems. Additionally, we identify putative recognition motifs for 24 additional Type III m4C systems from methylome analyses of SMRT sequenced genomes and identify hundreds of Type III systems highly similar to the characterized MTases that likely also depend upon m4C modification. These findings demonstrate Type III RM systems can employ either m6A or m4C for host protection, allowing even greater diversity of their recognition target as they evolve to defend against phage predation, along with an expanded potential for phase-variable transcription regulation.

## Materials and methods

### Materials

Restriction endonucleases, T4-DNA ligase, Phusion-HF and Q5 DNA polymerases, Gibson and HiFi assembly reagents, S-adenosylmethionine, Nucleoside Digestion Mix and competent *E*. *coli* cells were from New England Biolabs (Ipswich, MA, USA). Plasmid DNAs and PCR products were purified using spin-column reagents from New England Biolabs. DNA oligonucleotides (for use in PCR or DNA sequencing applications) and a codon-optimized synthetic gene were purchased from Integrated DNA Technologies (Coralville, IA, USA). Genomic DNAs of *D*. *thermolithotrophum* and *F*. *nodosum* Rt17-B1 were purchased from the DSMZ-German Collection of Microorganisms and Cell Cultures GmbH.

#### Identification of candidate Type III systems

A group of putative Type III RM systems that use m4C modification for host DNA protection were identified using Pacific Biosciences single-molecule real-time (SMRT) sequencing of bacterial genomic DNAs to identify asymmetric motifs having m4C methylation in one DNA strand [17]. SMRT sequencing was performed on the RSII platform and m4C methylation was characterized using the "RS_Modification_and_Motif_Analysis.1" program from the SMRT Analysis 2.3.0 software package. Raw sequence data is available from the SRA sequence read archives for four of the six strains described: *A*. *species* H (SRX10618618), *P*. *lemoignei* (SRX10565630 and SRX10565631), *E*. *coli* NCTC86 (SRX2568521) and *F*. *nodosum* Rt17-B1 (SRX1058899). Putative Type III RM systems were predicted using the SeqWare software in REBASE, based on sequence similarity between the predicted protein sequences and known Type III Mod and Res proteins.

#### Isolation of genomic DNAs

*A*. *species* H, *C*. *inhibens* subspecies *gilichinskyi* [20] and *P*. *lemoignei* genomic DNA samples were isolated using phenol-chloroform extraction followed by isopropanol precipitation. Genomic DNA of *E*. *coli* NCTC86 was a gift from Dr Swaine L. Chen.

#### Cloning and recombinant expression of *mod* genes

Candidate *mod* genes from each of the six bacterial strains were amplified from their respective genomic DNAs using Q5 high-fidelity DNA polymerase—and introduced into the high-copy pRRS plasmid vector using the HiFi *in vitro* DNA assembly method (NEB). DNA sequencing of the cloned genes and flanking vector sequences was used to confirm production of the *bona fide* recombinant plasmids. In the case of the *P*. *lemoignei mod* gene a non-canonical TTG initiation codon in the native strain was replaced with ATG for expression in *E*. *coli*. Cloned *mod* genes were expressed using either *E*. *coli* ER2683 (K strain, *dam*+/*dcm*+), *E*.*coli* C2523 (B strain, *dam*+/*dcm*-) or *E*. *coli* ER2796, a K strain which lacks all host-encoded methyltransferase activities [21].

#### Confirmation of methylation status of DNAs isolated from *E*. *coli* cells expressing Type III *mod* genes

Pacific Biosciences SMRT sequencing of plasmid and/or genomic DNA samples was used to confirm sites of incorporation of m4C modifications catalyzed by each of the Mod enzymes.

#### Quantitative analyses of *Mod* activities using an LC-MS assay

Relative abundances of unmethylated (dC) and methylated (m4C) were determined using liquid chromatography and mass spectrometry (LC-MS). In this case DNA samples were isolated from a *dcm*- *E*.*coli* host strain to eliminate m5C contamination which would otherwise confound the analyses. DNA samples were hydrolyzed to nucleosides using the Nucleoside Digestion Mix (NEB). LC-MS/MS analysis was performed in duplicate by injecting digested polynucleotide samples on an Agilent 1290 UHPLC equipped with a G4212A diode array detector and a 6490A Triple Quadrupole Mass Detector operating in the positive electrospray ionization mode. UHPLC was carried out using a Waters XSelect HSS T3 XP column (2.1 × 100 mm, 2.5 μm) with the gradient mobile phase consisting of methanol and 10 mM aqueous ammonium formate (pH 4.4). Data acquisition was performed in the dynamic multiple reaction monitoring (DMRM) mode. Each nucleoside was identified in the extracted chromatogram associated with its specific MS/MS transition: dC [M+H]^+^ at m/z 228 → 112 and m4C [M+H]^+^ at m/z 242 → 126. External calibration curves with known amounts of the nucleosides were used to calculate their ratios within the samples analyzed.

#### Restriction analyses

Two grams of *Escherichia coli* NCTC86 cells were suspended in 20 ml sonication buffer (20 mM Tris pH8.0, 50 mM NaCl, 0.1 mM EDTA), sonicated to create a cell extract and clarified by centrifugation at 30,000g for 15 minutes at 4°C. The extract was applied to a 5 ml Heparin HiTrap HP column equilibrated in sonication buffer and 3 ml fractions were collected during a 100 ml gradient from 50 mM to 1M NaCl in the same buffer. Fractions were assayed for endonuclease activity in 1X CutSmart buffer (NEB) supplemented with 1 mM ATP. A fraction containing Eco86II was identified and used to map cut sites on pUC19 and pBR322 plasmids. The Eco86II fraction was incubated with the closed circular plasmid DNA for 30 minutes, the reaction mixture was divided and one of several REases that cut each plasmid once at different positions around the plasmid was added and the reaction continued for 30 minutes more. DNA fragments were analyzed by electrophoresis on 1% agarose gels.

#### Sequence alignments

Protein sequence alignments of Type III restriction enzymes described in this study were constructed using PROMALS3D: a tool for multiple protein sequence and structure alignments [22].

## Results

**Fig 1:**
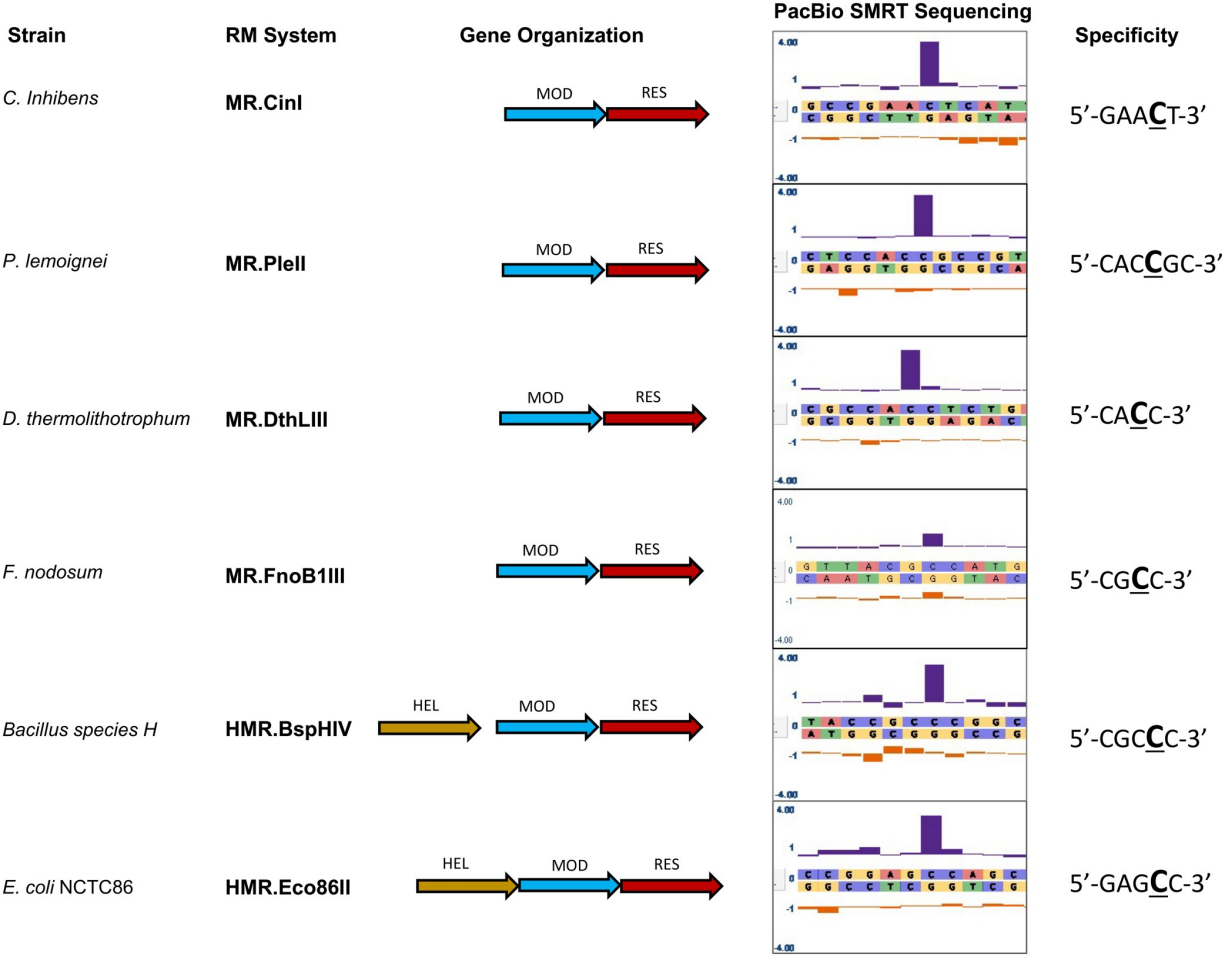
Identification of six Type III restriction-modification (RM) systems that methylate the N4 atoms of deoxycytosine bases. Four systems share a common architecture with previously-identified Type III systems comprised of Res and Mod subunits, whereas two additional systems appear to encode a third protein subunit located 5’ of their respective *mod* genes. Note that in the case of the BspHIV system there is approximately 140bp of non-coding sequence between the 3’-end of the helicase-like ATPase subunit and the 5’-end of the *mod* gene. SMRT-sequencing was employed to identify derived consensus methylation motifs for each of the six Type III RM systems. The tall blue bars indicate the Inter-Pulse Duration (IPD) values associated with positions of N4C methylation.

We characterized the recognition sequence motif, and the base modified to m4C within that motif, for six m4C Type III restriction-modification (RM) systems, each recognizing a novel target motif (Table 1). These systems were identified based upon observing an asymmetric, single-strand-modified motif typical of Type III RM systems, but having m4C methylation rather than m6A, in the SMRT sequencing methylome analyses of six diverged bacteria: *Carnobacterium inhibens* subspecies *gilichinskyi*, *Pseudomonas lemoignei*, *Desulfurobacterium thermolithotrophum*, *Fervidobacterium nodosum* Rt17-B1, *Eschericia coli* NCTC86 and *Acinetobacter species* H (formerly *Bacillus species* H, thus systems are named BspHI, BspHII, etc.) (Fig 1). Putative Type III RM system genes were identified within the genome sequences of these strains by a sequence similarity search comparing putative protein sequences to known Type III RM systems, using SeqWare software from the REBASE database [23]. Five of these six strains contained just one putative Type III system, while the *Acinetobacter species* H strain contained two putative Type III systems.

**Table 1:** Characterized m4C Type III R-M systems.

Enzyme	^a^Specificity	Host Organism	gDNA source
BspHIV	CGCCC	*Acinetobacter species* H.	NEB 394
CinI	GAACT	*Carnobacterium inhibens*	DSM 13024
DthLIII	CACC	*Desulfurobacterium thermolithotrophum*	DSM 11699
Eco86II	GAGCC	*Escherichia coli* NCTC86	Dr. Swaine L. Chen
FnoB1III	CGCC	*Fervidobacterium nodosum Rt17-B1*	DSM 5306
PleII	CACCGC	*Pseudomonas lemoignei*	NEB 418

^a^The cytosine base methylated in the recognition motif is underlined.

**Fig 2:**
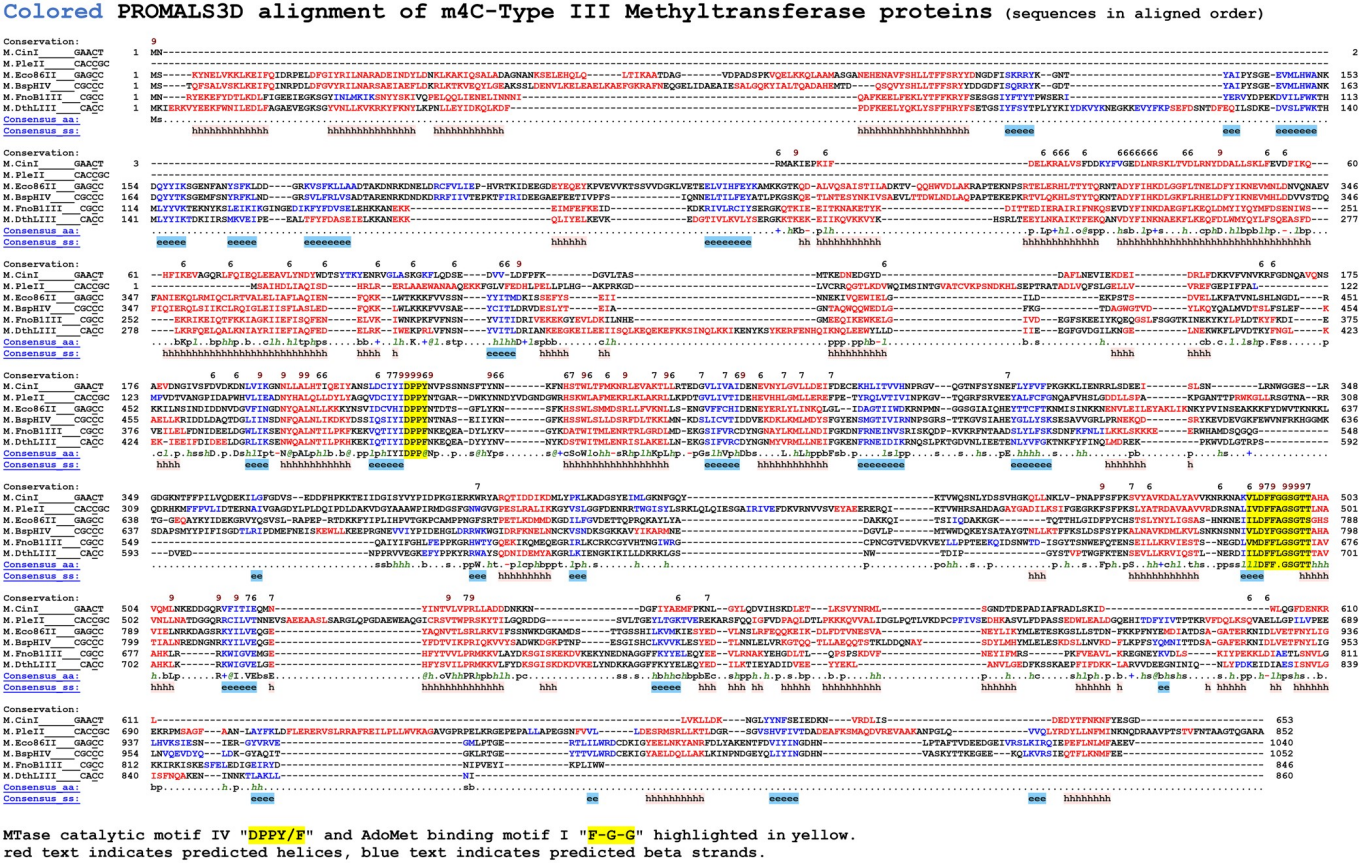
Alignment of the six characterized Type III Methyltransferases that methylate the N4 atom of cytosine. Each of the six Mod proteins are beta-class methyltransferases and contain conserved sequence motifs corresponding to the methyltransferase catalytic motif IV (DPPY) and the AdoMet binding motif I (FGG) described by Malone *et al*., [24].

**Fig 3:**
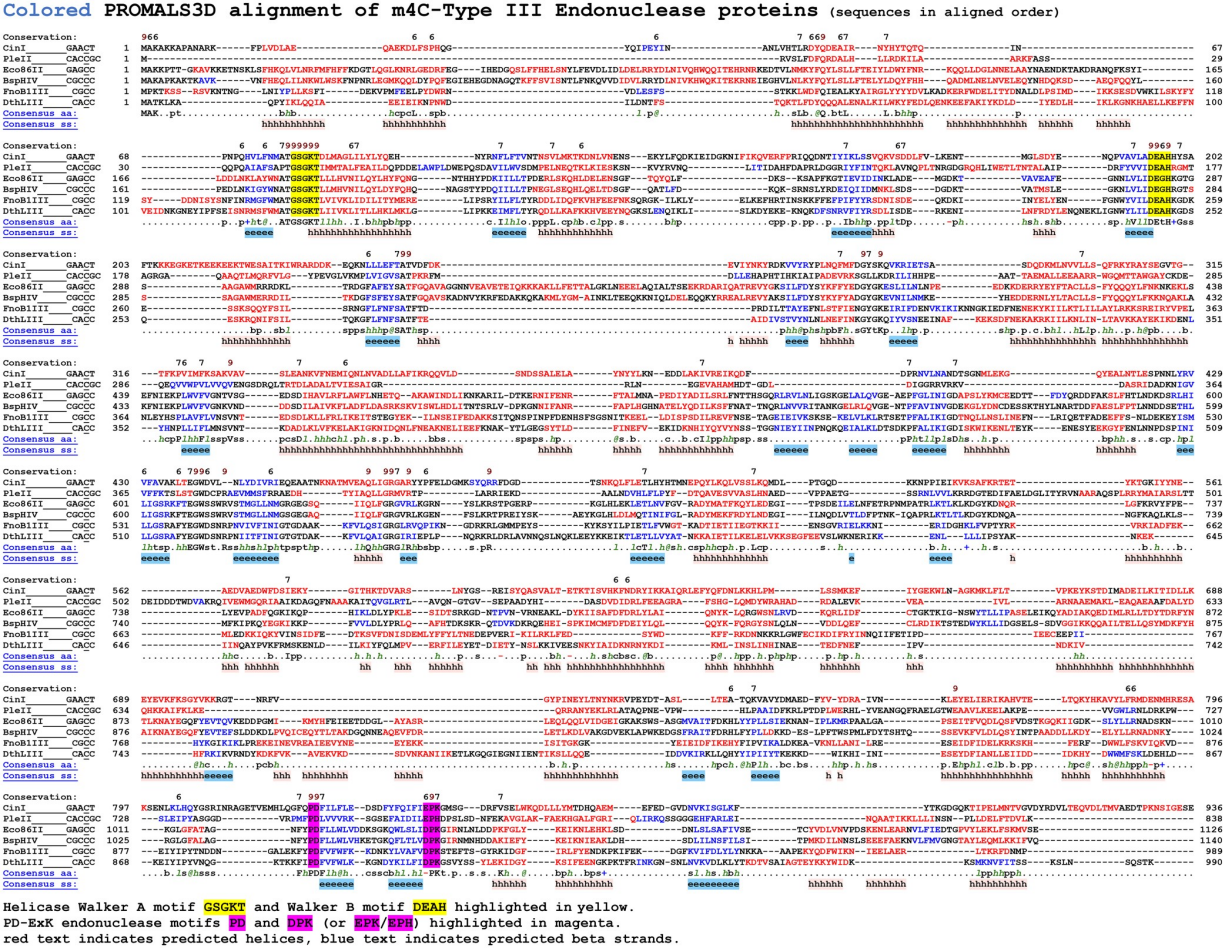
Alignment of the six characterized Type III endonucleases. The Res subunit of each system contains a conserved Walker A (GSGKT) and Walker B DEAD-BOX/DEXH motif typically associated with ATP-dependent DNA helicase proteins, and a canonical PD-(D/E)XK endonuclease catalytic motif located toward the carboxy terminus.

Each of these RM systems contains a Mod subunit with conserved amino-methyltransferase motifs, including the AdoMet binding motif I (FGG) and catalytic motif IV (DPPY/F) (Fig 2). Similarly, the Res subunit of each system contains conserved Walker A (GSGKT) and Walker B (DEXH) motifs typical of ATP-dependent helicase domains, and a canonical PD-(D/E)XK endonuclease catalytic motif in the carboxy terminal portion of the protein (Fig 3).

To match the identified specificity motif having m4C methylation to the putative Type III systems, and to identify which BspH system is responsible for the m4C-modified motif, each Mod gene, or both Mod plus Res gene, was PCR amplified, ligated into a suitable vector and transformed into an *E*. *coli* host. The transformed cells were grown to allow methylase expression and host modification, then total genomic and plasmid DNA was isolated and methylation motifs were identified using SMRT sequencing and methylome analyses.

### SMRT sequencing and LC-MS identification of m4C modification and recognition motifs

In contrast to all previously characterized Type III RM systems which form m6A, each of the newly identified systems methylate deoxycytosine bases yielding m4C. The methyltransferase of *C*. *inhibens* subspecies *gilichinskyi* (M.CinI) methylates the C at the fourth position of 5’-GAACT-3’ when expressed in *E*. *coli*, though the SMRT methylome analysis indicates methylation of this particular construct is partial (Table 2). The methyltransferase of *P*. *lemoignei* (M.PleII) methylates the C at the fourth position of 5’-CACCGC-3’. In this case expression of the Mod gene from the high copy plasmid pRRS resulted in full methylation of the natural target motif, CACCGC, but also additional methylation at near cognate sites representing relaxed specificity or ’star’ activity. The methylome analysis indicated significant methylation at CACCNC sites (Table 2), and lesser modification at CACCGT and CAACGC. This increased modification is likely a result of over-expression in a non-native context, as no modification of the extra star sites was observed in the native host. The methyltransferase of *E*. *coli NCTC86* (M.Eco86II) methylates the C at the fourth position of 5’-GAGCC-3’. Perhaps unsurprisingly, recombinant expression of this *E*. *coli* gene in our laboratory *E*. *coli* strain gave full methylation that was specific for the native site, even when expressed from the high copy vector, Table 2. We expressed the methyltransferase of both of the putative Type III systems found in *A*. *species* H and performed methylome analysis. M.BspHIV, locus tag HUK62_04085, was found to efficiently methylate the C at the fourth position of 5’-CGCCC-3’ when expressed in *E*. *coli*. The other putative Type III system from this strain was found to modify adenine to m6A in the recognition motif AGCACC and was designated BspHIII (MTase gene locus tag is HUK62_00355) Table 2. The *D*. *thermolithotrophum* and *F*. *nodosum Rt17-B1* systems (M.DthLIII and M.FnoB1III) were confirmed to methylate the Cs at the third position of 5’-CACC-3’ and 5’-CGCC-3’, respectively, the latter motif having been previously reported by Blow *et al*., [25].

**Table 2:** Modification detection by SMRT sequencing.

(dam GATC m6A reported for *E*. *coli* clones expressing dam MTase)				
Enzyme	MTase construct	^a^m4C % detected	Coverage	dam m6A %
BspHIV		CGCCC		
	Native organism	98.5	181x	na
	M.BspHIV-pRRS	97.6	90x	99.9
CinI		GAACT		
	Native organism	98.4	146x	na
	M.CinI-pRRS	25.3	82x	99.9
DthLIII		CACC		
	Native organism	85.5	550x	na
	M.DthLIII-pRRS	0	93x	99.9
	M.DthLIII+R.DthLIII-pRRS	90.6	2954x	100
Eco86II		GAGCC		
	Native organism	92.3	77x	99.6
	M.Eco86II-pRRS	98.9	76x	99.9
FnoB1III		CGCC		
	Native organism	47	not reported	na
	M.FnoB1III-pRRS	0	81x	99.9
	M.FnoB1III+R.FnoB1III-pRRS	63.7	163x	99.9
PleII		CACCGC		
	Native organism	99.9	158x	na
	M.PleII-pRRS	73.9	86x	99.9
	M.PleII-pRRS CACCNC "star"	69.3	86x	99.9

^a^"m4C percent detected" is percent of m4C motifs called as modified using the standard PacBio methylome analysis with default settings. Coverage is per strand for the motif. dam m6A methylation calls are shown as a control for systems expressed in *E*. *coli* carrying the dam methyltransferase.

Recombinant expression of the *mod* gene alone for M.BspHIV, M.CinI, M.Eco86II and M.PleII was sufficient to generate cytosine methylation in a laboratory *E*. *coli* strain lacking any endogenous methylation (ER2796). In addition to the SMRT sequencing methylome analyses, the m4C modified cytosine was detected via hydrolysis of the plasmid DNA to individual nucleosides, followed by quantitation using liquid chromatography and mass spectrometry (LC-MS), Table 3. Methylated cytosine residues were detected for these four systems expressing Mod alone. The percentage of modified C bases detected closely matched the expected value for full modification at the specific recognition motif for M.BspHIV and M.Eco86II (Table 3), in agreement with the SMRT methylome analysis detection of 97.6% and 98.9% of their sites as methylated (Table 2). An overabundance of modified cytosine relative to the number of target sites in the M.PleII expressing strain. Table 3 reflects the additional modification at near cognate ’star’ sites for this clone that is observed in the SMRT methylome analysis, Table 2. For M.CinI the LC-MS data showed only half the expected percentage of modified cytosine, which again agrees well with the SMRT methylome analysis that detected only partial modification.

**Table 3:** LC-MS analyses of m4C modification for Type III systems.

Enzyme	Genes	Specificity	m4C%	Target	Plasmid	Expected m4C%
			(m4C/m4C+C)	sites	size(kb)	sites/total "C"
BspHIV	mod	CGCCC	0.35%	9	5.9	0.36%
			0.35%			
CinI	mod	GAACT	0.21%	9	4.7	0.43%
			0.21%			
DthLIII	mod	CACC	0.0%	28	5.3	1.34%
			0.0%			
	mod+res		1.00%	32	8.3	1.11%
			1.00%			
FnoB1III	mod	CGCC	0.0%	31	5.3	1.48%
			0.0%			
	mod+res		1.31%	34	8.3	1.10%
			1.35%			
Eco86II	mod	GAGCC	0.23%	6	5.9	0.24%
			0.23%			
PleII	mod	CACCGC	1.24%	5	5.3	0.18%
			1.25%			

Plasmid DNAs expressing Type III *mod* genes were isolated from stationary phase cultures of *E*. *coli* C2523 (*dcm-/dam+*). Two independent isolates of each plasmid DNA were hydrolyzed to nucleosides using the Nucleoside Digestion Mix and resolved and quantified using LC-MS. m4C percentage methylation data indicate the mean values for each pair of independent plasmid replicates. Expected m4C percentage indicates the percentage of recognition motif cytosine targets in the substrate plasmid. The methylated bases from each isolate are highlighted in bold text. Note that in the case of the two thermophilic isolates co-expression of both *mod* and *res* genes is required for expression of their cognate MTase activities.

### Modification requirement for both MTase and ENase

In the case of the two systems isolated from thermophilic species, *D*. *thermolithotrophum* and *F*. *nodosum Rt17-B1*, we were unable to detect activity of the respective *mod* genes in *E*. *coli* using either SMRT methylome analyses or the LC-MS assay. Initially we assumed this was most likely a consequence of expressing the genes at a temperature much lower than optimal for the native strains, or alternatively, that the codon preferences of the thermophile genes were suboptimal for *E*. *coli* expression. In an attempt to address the latter, we obtained a synthetic *Desulfurobacterium mod* gene with a sequence that was codon optimized for expression in the heterologous *E*. *coli* host. However, when the synthetic gene was expressed in *E*. *coli*, we were still unable to detect any evidence of m4C modification by LC-MS, Table 3. This suggested that expression of the isolated *mod* gene alone was insufficient to confer methylation competency.

Although we are unaware of any precedent among previously characterized Type III systems, we decided to address the possibility that expression of the cognate *res* gene might be required to support methyltransferase activity. We therefore co-expressed the *mod* and *res* genes of both the *Desulfurobacterium* and *Fervidobacterium* RM systems in *E*. *coli* and in each case the previously absent m4C methyltransferase activity was detected upon co-expression of *mod* with the cognate *res* genes, Table 3.

### Restriction endonuclease activity

**Fig 4:**
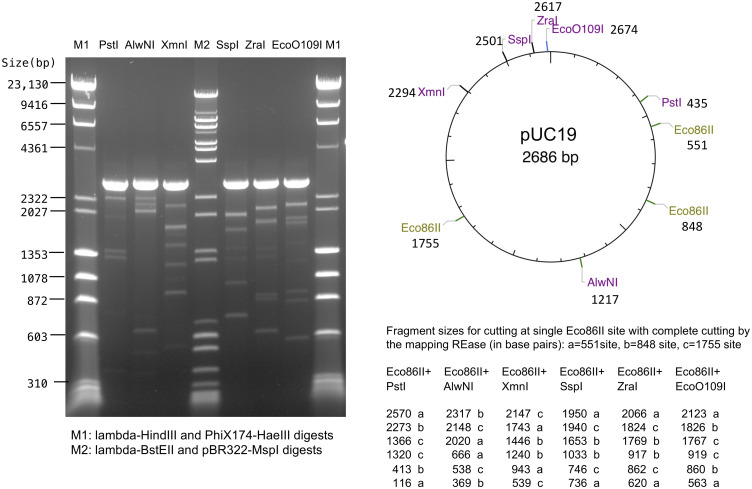
Eco86II endonuclease activity. Partially purified Eco86II enzyme was used to digest pUC19 plasmid substrate containing 3 sites: one in the forward direction and two in reverse orientation. After cutting with Eco86II, the reaction was divided and cut with six REases that cleave pUC19 once at known positions. Although the Eco86II digestion is only partial, fragments having lengths corresponding to the distance between the known REases and the Eco86II cleavage positions are observed, demonstrating specific Eco86II cleavage downstream from its GAGCC recognition motif. The original gel photograph is available as S1 Raw image.

To test if these systems have Type III endonuclease activity, we performed *in vitro* assays using a protein extract prepared from the Eco86II system native strain. We prepared a crude protein extract and partially purified this by ion exchange/affinity chromatography over a Heparin column. Heparin fractions were assayed for endonuclease activity by cutting either pUC19 or pBR322 plasmid DNA, both of which have head to head (HtH) oriented 5’-GAGCC-3’ Eco86II sites. After allowing the Type III system to act on the circular plasmid DNA, which enables 1D communication between HtH sites, the reaction was split and REases that cut once at various locations were added to linearize the plasmid and drop out diagnostic fragments generated by complete cutting at the linearizing REase site and any cutting by Eco86II. Although only partial Eco86II cutting was observed, the sites of cutting mapped to the expected positions for Eco86II (approximately 25 bp 3’ to the GAGCC Eco86II recognition sites in these DNAs), confirming that the Eco86II system has restriction as well as methylation function (Fig 4).

### Additional putative ATPase gene adjacent to MTase and ENase in some systems

The genomic organization for four of the new systems is similar to that of previously characterized Type III systems insofar as each contains a prototypical arrangement of *mod* and *res* genes. The remaining two systems differ in that they each have an additional, highly conserved gene located immediately 5’ of the *mod* gene and which has amino acid motifs typical of ATP-dependent SF2 DNA helicases. This potential extra helicase-like ATPase gene was not required for methyltransferase activity. We performed MS-MS peptide spectroscopy on the partially purified Eco86II restriction enzyme isolated from native cells (Fig 4) which detected both the mod and res proteins but no peptides from this putative additional helicase-like ATPase.

Type III systems that use m4C methylation are widespread. There are now 24 examples of Type III RM systems in REBASE [23] that can confidently be predicted to match a SMRT methylome m4C specificity motif. These enzymes recognize 17 different motifs and 5 different patterns of bases that are specifically recognized 5’ (2, 3 or 4 base pairs) and 3’ (1, 2 or 3 base pairs) to the cytosine that is methylated, Table 4.

**Table 4:** Putative m4C Type III R-M systems.

Enzyme	Specificity	Host Organism
Aha10306IV	CCACG	Acinetobacter haemolyticus NCTC10306
Bbr10542II	TCACCG	Bordetella bronchiseptica NCTC10542
Bca78520I	GAACCC	Brevibacterium casei 3012STDY7078520
Bhi13200II	CACCGC	Bordetella hinzii NCTC13200
Bpe564I	AGCCGCC	Bordetella pertussis H564
EcoF9792I	GAGCC	Escherichia coli F9792
EcoB4103II	GAGCC	Escherichia coli O27:H7 B4103-1
Gel16401II	GGACCG	Geopsychrobacter electrodiphilus DSM 15401
Kaq16071II	GGACT	Kangiella aquimarina DSM 16071
MspCY2I	AGCGCC	Microbacterium sp KROCY2
MspMM1I	CCACCC	Microbulbifer species CCB-MM1
Pmi29II	GAACT	Proteus mirabilis AR_0029
Sen6016III	GAGCC	Salmonella enterica NCTC6016
Sen10536IV	GAGCC	Salmonella enterica NCTC10436
TmeBIV	CGCC	Thermosipho melanesiensis BI429 DSM 12029
VflA21I	CARCCC	Vibrio fluvialis AK 1296-A2-1
Vna16374II	CCACCG	Vibrio natriegens CCUG 16374
Vsp20II	GCACCC	Vogesella species NEB_P20

Additional Type III m4C systems predicted from SMRT methylome data and bioinformatics as reported in the REBASE database. These additional Type III m4C systems are predicted from bacteria that have a single Type III system based on bioinformatic analysis of their predicted genes, and which have a single Type III-like single strand m4C modified motif detected in SMRT methylome analysis. While these systems have not been verified by biochemical assays or by expressing the system in a heterologous host and performing SMRT methylome analyses, the prediction of the recognition motif and function for these systems is of high confidence.

## Discussion

In this work we describe the first examples of Type III RM systems which exploit methylation of the N4 atoms of deoxycytosine for host DNA protection. Using SMRT sequencing of genomic DNAs we identified six candidate m4C Type III RM systems and defined the sequence specificities of their respective *mod* genes.

The m4C Type III systems characterized have rather diverse Mod protein sequences (Fig 2). This suggests either m4C methylation arose early in the evolution of Type III systems, or that it has arisen multiple times within diverged families of Type III systems. Since m6A and m4C MTases have identical chemistry in acting on an exocyclic nitrogen it seems likely that m4C modification could readily evolve from m6A MTases. The Res proteins are also quite diverse, also suggesting ancient lineage (Fig 3).

These six Type III RM systems characterized appear to fall into three groups. The *C*. *inhibens* and *P*. *lemoignei* group have a typical Type III architecture consisting of two genes, *mod* and *res*. In each case, recombinant expression of the respective *mod* gene alone was sufficient to confer robust m4C methyltransferase activity.

The second group is comprised of two systems isolated from the thermophilic bacteria *D*. *thermolithotrophum* and *F*. *nodosum Rt17-B1*. Robust methyltransferase activity was only detected in these systems when both the *mod* and *res* genes were co-expressed, suggesting that unlike other Type III systems mod alone is insufficient for methylation.

The third group of Type III systems, identified in the genomes of *A*. *species* H and *E*. *coli* NCTC86, differ from the other systems by virtue of an additional gene located 5’ of the *mod* and *res* genes. This additional gene contains sequence motifs typically associated with ATP-dependent helicase enzymes but does not contain a PD-ExK or another endonuclease motif. This putative ATPase is not homologous to the RES subunit of the adjacent Type III system. The biological role of this additional putative helicase-like ATPase gene is unclear although it appears to be intimately associated with its neighboring Type III system through the logic of guilt by association based on their consistently close proximity in more than 500 genomes. The Res subunits of Type III systems function by using the energy of ATP hydrolysis to rearrange the Mod2Res complex into a translocation competent complex that diffuses along the DNA to contact another Mod2Res complex bound at an inverted site. Whether this potential additional helicase-like ATPase supplements the translocase activities of the canonical Res subunit remains to be seen, but the intimate genomic association with the Mod and Res in these systems suggests this additional helicase-like ATPase plays some role in these systems. We examined the partially purified Eco86II enzyme preparation that gave partial cutting *in vitro* (Fig 4) using Mass Spec and did not observe any peptides from this additional helicase-like ATPase gene, indicating this extra protein is not required for Eco86II cleavage. Any role it may have will require further investigation.

Type III m6A methylation has been shown important in regulating gene expression and thus virulence through phase variable on-off expression in a number of bacteria [26]. The m4C Type III Mod systems extend this capability, but to date no examples have been described.

With these findings, m4C modification is now found across Type I, Type II and Type III RM systems. The Type I m4C systems have gamma-class MTases and all known such systems have one m6A and one m4C MTase protein and modify one strand to m6A and one to m4C. The Type I MTase proteins and their corresponding S recognition domains are highly similar, suggesting recent evolution of the m4C MTase from an m6A MTase ancestor. In contrast, the Type III m4C systems reported here are highly diverged and thus likely of ancient origin. Type II beta-class MTases are also quite diverged and likewise include enzymes that methylate either m6A or m4C. SMRT methylome analyses strongly suggest m4C modification also functions in further bacterial defense systems such as BREX systems.

## Supporting information

S1 File Raw sequence data accession numbers. (DOCX)

S1 Raw image (PDF)
