# Supplementary material for: Structural and functional diversity among Type III restriction-modification systems that confer host DNA protection via methylation of the N4 atom of cytosine
Source: PLoS One. 2021 Jul 6;16(7):e0253267. doi: 10.1371/journal.pone.0253267 (PMC8259958; doi:10.1371/journal.pone.0253267)
Supplement: S1 File — (DOCX) [file pone.0253267.s001.docx]

**S1 File.** SRA sequence read archives for four of the six strains described: *A. species* H (SRX10618618), *P. lemoignei* (SRX10565630 and SRX10565631), *E. coli* NCTC86 (SRX2568521) and *F. nodosum* Rt17-B1 (SRX1058899). Two strains were sequenced by others and not deposited so we cannot supply raw read archives.
