# Supplementary material for: Structural and functional diversity among Type III restriction-modification systems that confer host DNA protection via methylation of the N4 atom of cytosine
Source: PLoS One. 2021 Jul 6;16(7):e0253267. doi: 10.1371/journal.pone.0253267 (PMC8259958; doi:10.1371/journal.pone.0253267)
Supplement: S1 Raw image — (PDF) [file pone.0253267.s002.pdf]

Raw gel image for Fig 4. Eco86II endonuclease mapping on pUC19 DNA

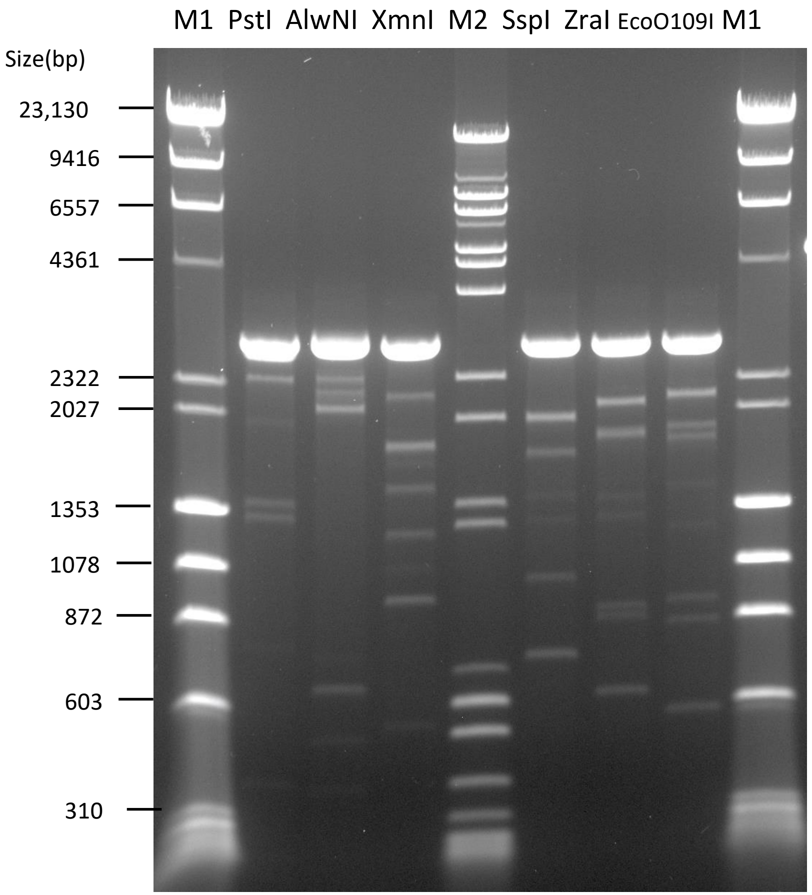

M1: lambda-HindIII and PhiX174-HaeIII digests.
M2: lambda-BstEII and pBR322-MspI digests.

DNA fragments were visualized using ethidium bromide staining and a UV transilluminator.
